# Prevalence and correlates of influenza-a in piggery workers and pigs in two communities in Lagos, Nigeria

**DOI:** 10.11604/pamj.2013.16.102.1450

**Published:** 2013-11-17

**Authors:** Emmanuel Jolaoluwa Awosanya, Gabriel Ogundipe, Olutayo Babalobi, Sunday Omilabu

**Affiliations:** 1 Department of Veterinary Public Health and Preventive Medicine, Faculty of Veterinary Medicine, University of Ibadan, Ibadan, Nigeria; 2 Department of Medical Microbiology and Parasitology, College of Medicine, University of Lagos, Idi-Araba, Lagos, Nigeria

**Keywords:** Prevalence, Influenza-A, piggery workers, pigs, practices, pig farmers, farm attendants, butchers, symptoms, Lagos

## Abstract

**Introduction:**

Worldwide, three Influenza-A virus subtypes (H1N1, H1N2 and H3N2) in swine are major public health issues. In Nigeria, the existence of these subtypes in pigs has not been well studied. This study aimed at determining the prevalence and correlates of Influenza-A viruses circulating in piggery workers and pigs in Oke-aro and Goshen communities in Lagos, Nigeria.

**Methods:**

Nasal swabs were taken from 197 consenting piggery workers and 281 randomly selected pigs to determine the prevalence of Influenza-A (H1, H3, H5) using Reverse Transcriptase Polymerase Chain Reaction test (gene M). An interviewer administered questionnaire was used to collect information on demography, Influenza-A related symptoms experienced, personal hygiene and management practices from the piggery workers. Descriptive statistics was used and chi square test performed at 5% significant level.

**Results:**

All piggery workers and pigs’ nasal swabs tested negative for Influenza-A viruses, hence, association could not be tested. Mean age of piggery workers was 41 ± 13.6 years and 60% were females. Forty two percent were farm attendants, 38.0% were pig farmers and the rest butchers. Nineteen percent had history of headache; 14.0% had catarrh and cough; 4.0% had sore-throat; 5.0% had diarrhea; while 48.0% had muscle pain at the time of data collection. The mean body temperature for the pig workers was 36.5 ± 0.5 °C. A significant difference (p<0.05) existed among piggery workers who had muscle pains.

**Conclusion:**

Piggery workers and pigs in study area were free of Influenza-A (H1, H3, H5) viruses. The current practices of the piggery workers should be encouraged.

## Background

The pandemic of Influenza-A H1NI that hit the world especially the Asian continent in 2009 caused a serious public health scare. It also caused social threats especially among piggery workers and led to a downward trend in pig farming activities and businesses [1, 2]. The 1918 Influenza pandemic in humans was associated with H1N1 and Influenza appearing in pigs, an experience similar to that of 2009 pandemic Influenza outbreak [3, 4]. Certain studies have also documented the role of the pigs in Influenza-A epidemiology [5–7].

Several subtypes of Influenza-A virus have been found to infect pigs under experimental and field conditions [8–10]. Three Influenza-A virus subtypes – H1N1, H3N2 and H1N2 – are currently circulating in swine worldwide, but the origins of the antigenic and genetic characteristics of these swine Influenza virus subtypes differ in different continents or regions of the world [8]. Pigs serve as major reservoirs of H1N1 and H3N2 Influenza viruses and are often involved in interspecies transmission of Influenza viruses [11]. The maintenance of these viruses in pigs and the frequent introduction of new viruses from other species could be important in the generation of pandemic strains of human influenza.

Algeria, Cote d’ Ivoire, Egypt, Ethiopia, Morocco, South Africa, and Tunisia have reported cases of the new pandemic Influenza-A H1N1 virus; Nigeria reported its first case of the pandemic Influenza-A H1N1 virus in November 2009 and had also reported outbreaks of highly pathogenic Avian Influenza H5N1 in poultry and one case of human infection in 2008. Lagos State, one of the 36 States in Nigeria, has a history of highly pathogenic Avian Influenza outbreaks in birds and humans and also reported seven out of the nine suspected cases of H1N1 in Nigeria with one fatality in 2009.

In 2008 some Influenza-A subtypes (which were H1N1, H3N2 and A (H1N1)-A(H3N2) double reactant) were isolated from apparently health pigs at a farm in Ibadan, Nigeria [12]. However, the burden of Influenza-A among piggery workers and pigs in Lagos, Nigeria is unknown.

We set out to determine the magnitude and the correlates of Influenza-A viruses circulating among piggery workers and pigs in Oke-Aro and Goshen communities of Lagos from April to June 2010, so as to provide information that will guide policy making in the preparedness, resource allocation, control and prevention of Influenza-A infection.

## Methods

**Study Design:** We conducted a cross sectional study among piggery workers and pigs at selected slaughter slabs and pig farms in Lagos State, Nigeria from April to June, 2010.

**Study sites** The study sites were Oke-aro and Goshen communities of Lagos State, South west Nigeria. Lagos State has 20 Local Government Areas (LGAs) with a total population of 9,013,534 people and male: female ratio of 1.079:1 [13]. Oke-aro and Goshen are respectively in Ifako/ Ijaye and Agege LGAs of Lagos State. Ifako/ Ijaye LGA in 2006 had a population of 427,878 and Agege LGA had a population of 459,939 [14]. The pig population in Lagos could not be ascertained; however it was estimated to be around one million pigs. Lagos State has several pig farms, an abattoir and eight government-recognized slaughter houses; pigs are slaughtered in only two of these slaughter houses – Goshen and Oke-aro. Oke-aro is the largest pig farm estate in Lagos State with various cooperative societies. Lagos State is the epicenter of pig farming activities in Nigeria with history of Avian and pandemic Influenza-A.

**Sample size and sampling:** A total sample size of 197 piggery workers and 281 pigs were involved in the study. We approached this by using the formula below:

n = Z^2^ p (1 - p)/d^2^

Where Z is the reliability coefficient put at 1.96 at 95% confidence intervals. Based on previous studies we powered the study to detect prevalence (p) in humans of 10.9% [7]; while in pigs, prevalence of 20.5% was used [15]. The precision (d) was 5%. The calculation gave a minimum sample size of 150 and 255 for piggery workers and pigs respectively. Addition of 31% and 10% non-response rate gave the final sample size of 197 and 281 for piggery workers and pigs respectively. We used 31% non response rate for human participants based on some of the initial challenges we encountered with recruiting: initial consideration was 10%, however, in other to accommodate principles of research ethic that borders on respect for persons and justice; 17 participants declined of the original 165 participants obtained after considering 10% non-response rate. The 17 participants who declined were from the first two sets of farmers’ cooperative societies approached, however, after intensified sensitization and beneficence 49 other piggery workers that were approached indicated interest in the study and were thus recruited.

The pigs used for this study were pigs to be slaughtered at the study sites. The pigs were selected by systematic random sampling at a sampling interval of 2 from an estimated number of pigs slaughtered per day (50). Twenty pigs were sampled per day. The pig farms and the pig farmers and attendants were selected by simple random sampling from lists that we obtained from the pig farmers’ cooperative societies. The butchers were also selected by simple random sampling from lists that we obtained from the butchers’ cooperative society at the study sites. Informed consent was obtained from all human participants. Piggery workers who declined to participate were excluded from the study.

### Data collection

**Nasal swab collection:** Nasal swabs were taken from both the piggery workers (197) and pigs (281). The nasal swabs were obtained by inserting sterile nasal swabs into the nares for few seconds and removed by rotatory movement down the sides of the nares. One swab was used for both nares. The swabs were preserved in sterile tubes containing virus transporting medium and sent in ice packs to the laboratories for analysis.

**Blood collection:** We obtained venous blood (5mls) from all the piggery workers and exsanguinated blood from sampled pigs for serology into sterile plain sample bottles and sent in ice packs to the laboratories for analysis.

**Questionnaire:** A semi-structured, interviewer administered questionnaire was used to obtain information on piggery workers’ demography, Influenza-like symptoms experienced at data collection time, personal hygiene and management practices. We used the mercury thermometer to obtain the body temperature in degree centigrade of participating piggery workers.

### Laboratory analysis

The human samples were analyzed within 48 hours after collection at the Central Research Laboratory, Department of Medical Microbiology and Parasitology, College of Medicine, University of Lagos. The animal samples were analyzed about four weeks after collection at the National Veterinary Research Institute, Vom-Jos, Plateau State, Nigeria. One Step Reverse Transcriptase Polymerase Chain Reaction (RT-PCR) test was performed.

**RNA extraction:** RNA was extracted from 140 µL of nasal swab sample using a QIAamp viral RNA extraction kit (QIAGEN Inc., Valencia, CA, USA). The extraction was done according to the manufacturer's instructions. Inactivated H5N2 antigen from World Organization for Animal Health (OIE)/ Food and Agriculture Organization (FAO) reference laboratory in Padova, Italy was used as positive controls while nuclease-free water was used as negative control. Negative controls and positive controls were included in each extraction run.

**Screening for Influenza A:** Influenza-A was screened for using the protocol described by Fouchiers and others [16] – one step RT-PCR Influenza type A. Negative template controls and positive controls were included in each run.

**Gene amplification:** This was done as described by Fouchiers and others [16].

**Serology:** We did Enzyme Linked Immunosorbent Assay (ELISA) test using Influenza-A IgG ELISA kit for humans -Beijing and Sydney strains (BioSupply UK Limited) and Swine Influenza ELISA kit (ID Vet innovative diagnostic, France) to screen both human and animal samples for antibodies to Influenza-A.

**Data analysis:** Percentages, means and standard deviations were determined using Microsoft Excel, 2003. Statistical significance was determined using Chi squares test at 95% confidence intervals using Epi-Info version 3.3. Probability level of less than 5% (P<0.05) was accepted as significant.

## Results

**Figure 1:**
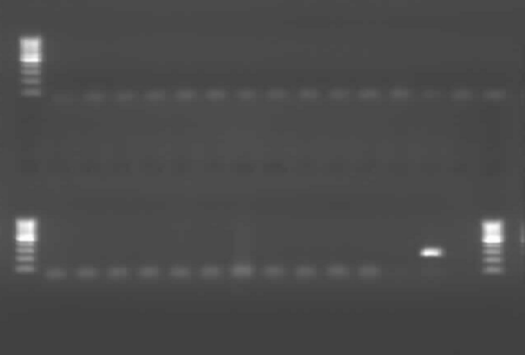
RNA Gel Electrophoresis Image of the pig nasal swab extracts showing their negativity for Influenza-A virus

All the 197 human nasal swabs and 281 pig nasal swabs collected and tested were negative to Influenza-A viruses by RT-PCR (Figure 1). Of the 197 piggery workers and 281 pig sera screened for Influenza-A by ELISA 171 (87%) and 188 (67%) were positive respectively.

A total of 197 piggery workers responded: 42% were farm attendants, 38% were pig farmers while the rest were butchers. The mean age of respondents was 41(Standard Deviation (SD) 13.6) years. The farmers had the highest mean age of 50.2 (SD 12.8) years (Table 1). All had normal (36.5 (SD 0.5) 0C) body temperature. Sixty percent of the respondents were female. More males were farmers than females; while more females were farm attendants than males (Table 1).

**Table 1:** Demographic features and Influenza-A related symptoms experienced by farmers, butchers, and attendants at time of data collection at Oke-aro and Goshen communities, Lagos State

PARAMETERS	FARMERS ( n=75)	BUTCHERS (n=40)	ATTENDANTS (n=82)
**Mean temp (**^**°**^**C)**	36.4 ± 0.5	36.5 ± 0.4	36.5 ± 0.6
**Mean Age (yrs.)**	50.2 ± 12.8	35.6 ± 10.7	35.3 ± 10.9
**Gender ratio f/m)**	26/ 49 (1:2)	18/ 22 (1:1)	74/ 8 (9:1)
**Symptoms**			
**Catarrh**	10 (13.3%)	7 (17.5%)	10 (12.2%)
**Sore throat**	2 (2.7%)	3 (7.5%)	3 (3.7%)
**Cough**	10 (13.3%)	4 (10%)	13 (15.9%)
**Headache**	14 (18.7%)	8 (20%)	15 (18.3%)
**Diarrhea**	5 (6.7%)	2 (5%)	2 (2.4%)
**Muscle pain**	29 (38.7%)	26 (65%)	39 (47.6%)*

* N.B: Significant at p<0.05

At the time of data collection, respondents had the following Influenza-like symptoms: 48% had muscle pain, 19% had headache; 14% had catarrh and cough each; 5% had diarrhea; while 4% had sore-throat (Table 1). The least reported Influenza-like symptoms were sore throat among farmers and diarrhea in both butchers and farm attendants (Table 1).

Most (85%) of the piggery workers reported they took a bath after work. Only 16% of the piggery workers used either hand gloves or protective boots, however, all had change of clothing before and after work. Only 35% of the piggery workers reported washing hands with soap and water before eating. There was a significant difference in the percentage sero-positivity between farmers and butchers (Table 2). Piggery workers who do not use personal protective equipment (PPE) (i.e., gloves and boots) were 4 times more likely to be sero-positive to Influenza-A than those who do (Table 2).

**Table 2:** Correlates of Influenza-A sero-positivity among Piggery workers at Oke-aro and Goshen Communities, Lagos State, 2010

Variables	Sero-positive (n=171)	Sero-negative (n=26)	Prevalence Odds Ratio	95% Confidence Interval	P Value
**Gender**					0.64
Male	67 (39.2%)	12 (46.2%)	0.75	0.30 – 1.86	
Female	104 (60.8%)	14 (53.8%)	1.33	0.54 – 3.28
**Designation**					
Farmers	61 (81.3%)	14 (18.7%)	Ref.		
Attendants	70 (85.4%)	12 (14.6%)	0.75	0.30 – 1. 88	0.64
Butchers	40 (100.0%)	0 (0.0)	0.00	0.00 – 0.59	0.002*
**Hygiene Practices**					
Use of PPE	22 (12.9%)	10 (38.5%)	0.24	0.09 – 0.64	0.003*
Do not use PPE	149 (87.1%)	16 (61.5%)	4.23	1.55 – 11.49	
Taking of bath after work	142 (83.0%)	25 (96.2%)	0.20	0.01 – 1.45	0.14
Washing of hands with soap before eating	56 (32.8%)	13 (50.0%)	0.49	0.20 – 1.21	0.13

Data are frequencies of sero-positive and sero-negative piggery workers by gender, designation and hygiene practices (%) with their odds ratios (OR), 95% confidence interval (CI) and Probability value (P).

* Significant

All of the pig farmers had 4 to 6 growers which are any pig between weaning (i.e., 6 – 8 weeks of age) and sale or transfer to the breeding herd, sold for slaughter or killed for rations (about 6 months of age) – per compartment. All pig farmers practiced the production policy of All-in All-out system which is a batch system of pig rearing. All pig farmers reported the presence of disinfectants at the main entrance to the pig estate and checking of movement into and out of the pig estate. All pig farmers reared only pigs and all re-stocked their pens by sourcing from within the pig estate. Butchers from Goshen reported to have obtained the pigs they slaughtered from various pig farms within Lagos State. Most (83%) of the butchers used their teeth in removing pig hooves during processing.

## Discussion

We report in our study of two communities in Lagos which is the epi-center of pig farming in Nigeria Influenza-A sero-prevalence of 87% among piggery workers and 67% in pigs by ELISA which indicates previous exposure to Influenza-A. We also report a prevalence of zero percent to Influenza-A by RT-PCR in both piggery workers and pigs in these two communities which indicates a possibility of an absence of recent exposure at the time of sample collection.

The high sero-prevalence of Influenza-A in our study is similar to that reported by Lopez-Robles and others who reported high sero-prevalence of Influenza-A among piggery workers in Mexico and Luiz et al who reported sero-prevalence of 46% and 20% for Influenza-A H3N2 in pig farms and among pigs in Parana, South Brazil using haemagluttinin inhibition assay [17, 18]. Vaccination against any of the Influenzas (i.e., Seasonal, Zoonotic, Pandemic) is not practiced in Nigeria thus the presence of IgG antibodies to Influenza-A in both human and animal samples is suggestive of a real time exposure to any of Influenza-A field strain viruses.

Our finding of zero percent prevalence for Influenz-A by RT-PCR in both piggery workers and pigs in the two communities despite high sero-prevalence is similar to that reported by Suriya and others [19] who reported sero-prevalence of swine Influenza H1N1 and H3N2 among pigs in Peninsular, Malaysia to be 12.2% and 12.1% respectively by ELISA. However, no virus or viral nucleic acid (zero prevalence) was detected from nasal swabs of sero-positive pigs by virus isolation and real time RT-PC. The absence of fever in all of the piggery workers and the lower frequency in reported Influenza-like symptoms supported the possibility of absence of recent infection among the piggery workers.

Seasonality could also be another reason for the zero prevalence among the piggery workers and pigs. Although it has been reported that the Influenza-A virus can circulate among pigs throughout the year, there is a seasonal pattern to Influenza in humans and pigs [20]. Influenza-A virus has been reported to thrive well in low ambient relative humidity and low ambient temperature and to lose viability in relatively high ambient humidity and high environmental temperature [21]. The high environmental temperature at the study site which was about 31° Centrigade and the period of sample collection which was at the transition phase between dry and rainy season could be the reason for the zero prevalence. Ambient relative humidity between 50 and 84% and environmental temperature about 30°Centrigrade make Influenza-A virus unviable [21, 22].

This study revealed that movement in and out of the Oke-aro farm estate was checked and that disinfectants were positioned at the main entrance to the farm estate. Also that each compartment in a pen contained 4 to 6 growers; and that replacement stocks are sourced within – no farmer is allowed to bring in new stock of pigs from outside the pig estate. Only pigs were raised on the farm estate and All-in All-out policy was practiced – a batch system of pig farming. These factors could have contributed to the zero prevalence of Influenza-A in the sampled pigs from this farming estate. This is similar to the findings of Poljak and others [23] who reported that factors associated with sow-herd H1N1 positivity included pig or farm density, an external sources of breeding pigs, number of animals on site and decreasing proximity to other barns. Herd size was reported to be positively associated with an increased probability of respiratory disease [24]. The above observation is also supported by Easterday and Van [20] who reported that the most important factor for incident cases of Influenza at the farm level is the introduction of animals carrying the infection into a naïve herd. In contrast, Suriya and others [19] reported significant association between sero-positivity of pigs to H1N1 and H3N2 based on ELISA and factors such as size of farms; importation or purchase of pigs and presence of mammalian pets within the farm – factors that were not reported by our study participants.

We found that farmers had a lower sero-prevalence (81.3%) of Influenza-A antibodies than the butchers (100%). The difference was statistically significant (OR = 0.00, 95% CI 0.00 – 0.59). This is in contrast to the findings of Myers and others [7] who reported highest exposure to Swine H1N1 for farmers and least among meat processing worker (butcher). The reasons could be that most pig farmers in the communities that we studied play more role of a supervisor than actual involvement in the farming work and that some of the butchers engaged in some unhygienic practices like removal of pig hooves with their teeth during processing. The reasons adduced by Myers and others [7] for lower risk of exposure to Swine H1N1 in slaughter house workers than farmers and veterinarians was the rarity of viraemia in pigs and that highest concentration of virus would be in the lungs and respiratory tissue and not in the intestinal tract. However, butchers in our study areas do not only involve in slaughtering of pigs but also have exposures to pigs before they are slaughtered and to the visceral after slaughtering.

The majority of the farmers, farm attendants and butchers did not use PPE such as safety boots and hand gloves. However all reported that they change their clothes before and after working as well as take a bath after work except for some farmers who play supervisory role. The latter factor may also explain the zero prevalence among the piggery workers. Teleman and others [25] showed that hand washing and use of mask can reduce the transmission of Influenza in health care settings. The use of PPE such as safety boots and hand gloves was found to be associated with a low prevalence of Influenza-A by ELISA. It has been reported that piggery workers’ risk of high antibody titer to swine H1N1 virus was reduced almost to that of non-exposed controls if the piggery workers reported using gloves [26]. The use of PPE has been reported to be helpful in prevention and control of Influenza-A [27]. This is also similar to the findings of Morgan and others [28]; however, Toyokawa and others [29] reported no significant difference in pandemic Influenza H1N1 sero-positive rate for any PPE among Health care workers in Japan.

Generalization of the findings from our study is subject to at least two limitations. Firstly, the specific subtype of the Swine Influenza ELISA kit (ID Vet innovative diagnostic, France) used was not indicated by the manufacturer thus we could not ascertain which particular subtypes circulate among the exposed individuals. Secondly, the study could not be extended for a whole year to accommodate seasonal variations for time constraints. However, these limitations were considered in the interpretations of the study findings.

Our study revealed a strong evidence of previous exposure to Influenza-A viruses among the piggery workers and pigs in this study sites in 2010, though evidence of recent exposure could not be ascertained. Influenza-A viruses have high burden among piggery workers and pigs in the study sites. Seasonal influence, high environmental temperature at the time of sample collection, farm bio-security, farm management of all in, all out policy and internal sourcing of replacement stock for pigs might have contributed to lack of evidence of recent exposure among the piggery workers and pigs at the study sites. Regular use of PPE is associated with being sero-negative for Influenza-A among the piggery workers.

In view of the study findings, we recommended that the regular use of PPE should be encouraged among the piggery workers and practices of farm bio-security, farm management of all in all out policy and internal sourcing of replacement stock for pigs should be encouraged among the pig farms. A study on prevalence (serology and RT-PCR) and correlates of Influenza-A among piggery workers which will cover a whole year to take care of possible seasonal variations should be carried out.

## Conclusions

Piggery workers and pigs in this study sites were free of Influenza A (H1, H3, H5) virus, this may be due to seasonal influence. The piggery workers were adjudged free of Influenza despite some having influenza-like symptoms because none had fever. The high environmental temperature at the time of sample collection, seasonal variation, long storage period between sample collection and sample laboratory analysis, having regular bath after work and change of cloth, farm bio-security, farm management of all in, all out policy and internal sourcing of replacement stock for pigs might have contributed to the zero prevalence among the piggery workers and pigs at the study sites. In view of the study findings, it is recommended that the current practices of having regular bath after work and change of cloth, farm bio-security, farm management of all in, all out policy and internal sourcing of replacement stock for pigs should be encouraged among the piggery workers and that more than one test should be employed for Influenza virus detection. A study on prevalence (serology and RT-PCR) and correlates of Influenza-A among piggery workers which will cover a whole year to take care of possible seasonal variations should be carried out.
